# 新型功能材料在藻毒素萃取中的应用进展

**DOI:** 10.3724/SP.J.1123.2023.10006

**Published:** 2024-03-08

**Authors:** Min FANG, Yaping WU, Wenmin ZHANG, Lan ZHANG, Zhenquan YANG

**Affiliations:** 1.扬州大学食品科学与工程学院, 江苏 扬州 225127; 1. College of Food Science and Engineering, Yangzhou University, Yangzhou 225127, China; 2.福州大学化学学院, 福建 福州 350116; 2. College of Chemistry, Fuzhou University, Fuzhou 350116, China; 3.闽江师范高等专科学校化学与生物技术学院, 福建 福州 350108; 3. Department of Chemistry and Biotechnology, Minjiang Teachers College, Fuzhou 350108, China

**Keywords:** 碳基材料, 金属有机骨架, 共价有机骨架, 分子印迹聚合物, 藻毒素, 综述, carbon-based material, metal organic framework (MOF), covalent organic framework (COF), molecularly imprinted polymer, algal toxins, review

## Abstract

藻毒素为有害藻类所产生的次级代谢产物,具有毒性强、种类多和生物蓄积性等特点,对人类健康、水产养殖业以及水生生态系统都会造成严重的威胁,已成为当前全球范围的研究热点。由于藻毒素在样品中的含量很低、样品基质复杂等因素,在仪器分析前进行有效的样品前处理不可或缺。高效的样品前处理技术不但能够减小或去除样品基质对分析的干扰,而且可以实现目标物的富集,增加分析方法的灵敏度与准确性。近年来,固相萃取(SPE)、固相微萃取(SPME)、磁性固相萃取(MSPE)、分散固相萃取(DSPE)、吸管尖端固相萃取(PT-SPE)等样品前处理技术已在藻毒素分离分析领域广受关注。这些前处理技术性能的好坏主要取决于萃取材料的特性。由于藻毒素的理化特性各不相同,在分子尺寸、亲疏水性、电荷等性质上差异较大,合理设计并制备适合藻毒素萃取的材料十分必要。最佳的萃取材料必须实现对藻毒素的可逆吸附,并且最好具有多孔结构和高的比表面积,从而能够提供高的回收率和与藻毒素良好的界面接触。此外,萃取材料还应该在样品溶液、洗脱溶剂、工作pH范围内具有良好的化学稳定性,否则萃取材料可能会溶解或丢失其官能团。本文综述了近十几年来国内外关于藻毒素分析检测研究的主要代表性文献,对新型功能材料在藻毒素萃取过程中的应用进行了梳理归纳,并对其发展前景予以展望。

## Abstract (en)

Algal toxins are secondary metabolites produced by harmful algae; these metabolites are characterized with strong toxicity, diverse structure and bioaccumulation. Aquatic organisms that feed on harmful algae can accumulate algal toxins in their bodies, and the consumption of these organisms by humans can cause symptoms of paralysis, diarrhea, and even death. The onset of poisoning can occur within as little as 30 min; in many cases, no suitable antidote for algal toxins is available. Thus, algal toxins present significant threats to human health, the aquaculture industry, and aquatic ecosystems. Because the potential risks of algal toxins are a critical issue, these toxins have become a research hotspot. The water environment and various types of aquatic products should be monitored and analyzed to ensure their safety. However, because of possible matrix effects and the low content of algal toxins in actual samples, an efficient pretreatment method is necessary prior to instrumental analyses. Efficient sample pretreatment techniques can not only reduce or eliminate interferences from the sample matrix during analysis but also enrich the target analytes to meet the detection limit of the analytical instrument, thereby ensuring the sensitivity and accuracy of the detection method. In recent years, sample pretreatment techniques such as solid-phase extraction (SPE), solid-phase microextraction (SPME), magnetic SPE (MSPE), dispersive SPE (DSPE), and pipette tip-based SPE (PT-SPE) have gained wide attention in the field of algal-toxin separation and analysis. The performance of these pretreatment techniques largely depends on the characteristics of the extraction materials. Given the diverse physicochemical properties of algal toxins, including their different molecular sizes, hydrophobicity/hydrophilicity, and charges, the design and preparation of materials suitable for algal-toxin extraction is an essential undertaking. The optimal extraction material should be capable of reversible algal-toxin adsorption and preferably possess a porous structure with a large surface area to allow for high recovery rates and good interfacial contact with the toxins. Additionally, the extraction material should exhibit good chemical stability in the sample solution and elution solvent within the working pH range; otherwise, it may dissolve or lose its functional groups. Many research efforts have sought to develop novel adsorbent materials with these properties in the separation and analysis of algal toxins, focusing on carbon-based materials, metal organic frameworks (MOFs), covalent organic frameworks (COFs), molecularly imprinted polymers (MIPs), and their functionalized counterparts. Carbon-based materials, MOFs, and COFs have advantages such as large surface areas and abundant adsorption sites. These extraction materials are widely used in the separation and analysis of target substances in complex environmental, biological, and food samples owing to their excellent performance and unique microstructure. They are also the main adsorbents used for the extraction of algal toxins. These extraction materials play an essential role in the extraction of algal toxins, but they also present a number of limitations: (1) Carbon-based materials, MOFs, and COFs have relatively poor selective-adsorption ability towards target substances; (2) Most MOFs are unstable in aqueous solutions and challenging to apply during extraction from water-based sample solutions; (3) COFs mainly consist of lightweight elements, rendering them difficult to completely separate from sample solutions using centrifugal force, which limits their application range; (4) Although MIPs have good selectivity, issues such as template-molecule loss, slow mass-transfer rates, and low adsorption capacity must be addressed. Therefore, the design and preparation of novel functionalized extraction materials specifically tailored for algal toxins and studies on new composite extraction materials are highly desirable. This article collects representative literature from domestic and international research on algal-toxin analysis over the past decade, summarizes the relevant findings, categorizes the applications of novel functional materials in algal-toxin-extraction processes, and provides an outlook on their future development prospects.

藻毒素是由有毒藻种产生的一类次级代谢产物,很容易通过食物链在贝类、鱼类等水产品中蓄积^[1]^。它们毒性强,可引起麻痹、腹泻,甚至死亡等中毒症状,发病时间最快可至30 min,无特效解毒剂,对消费者的健康危害极大^[2,3]^。产毒藻类主要以甲藻门(Pyrrophyta)、蓝藻门(Cyanophyta)、金藻门(Chrysophyta)为主^[4]^,它们产生的毒素结构多种多样,有生物碱、聚醚类化合物、肽类化合物、氨基酸等(见表1)。近年来,随着藻毒素污染范围的不断扩大,分析样品存在基质复杂、目标物含量低等特点,给分析检测带来了严峻的挑战,往往需要有效的样品前处理技术才能保证分析结果的可靠性和准确性。

**表 1:** 代表性藻毒素的信息

Phylum	Representative toxin	Type
Pyrrophyta	saxitoxin (STX)	alkaloid containing
		guanidine group
	gonyautoxin (GTX)	alkaloid
	brevetoxin (PbTX)	polyether compound
	ciguatoxin (CTX)	polyether compound
	okadaic acid (OA)	fatty acid polyether
		compound
	palytoxin (PTX)	polyether compound
Cyanophyta	microcystins (MCs)	heptapeptide
	nodularins (NODs)	pentapeptide
Chrysophyta	domoic acid (DA)	amino acid

目前,常见的藻毒素前处理技术有固相萃取(solid-phase extraction, SPE)、固相微萃取(solid-phase microextraction, SPME)、磁性固相萃取(magnetic SPE, MSPE)、分散固相萃取(dispersive SPE, DSPE)、吸管尖端固相萃取(pipette tip-based SPE, PT-SPE)等^[5]^。其中,萃取材料是这些方法的重要组成部分,直接影响了萃取方法的性能。由于藻毒素的理化特性各不相同,在分子尺寸、亲疏水性、电荷等性质上差异较大,合理设计并制备适合于藻毒素萃取的材料十分必要。最佳的萃取材料必须实现对藻毒素的可逆吸附,且最好具有多孔结构和高比表面积,从而能够提供高的回收率和与藻毒素良好的界面接触。此外,萃取材料还应该在样品溶液、洗脱溶剂、工作pH范围内具有良好的化学稳定性,否则萃取材料可能会溶解或丢失其官能团。本文梳理归纳了近十几年来国内外关于藻毒素分析检测研究的主要代表性文献,对新型功能材料在藻毒素萃取过程中的应用进行了评述,并对其发展前景予以展望。

## 1 碳基材料

碳基材料(carbon-based material)凭借高比表面积、优异的吸附性能、易修饰性等优点,已被广泛应用于储能、催化、分离富集和环境修复等领域。常见的碳基材料包括石墨烯、氧化石墨烯(GO)、碳纳米管(CNT)等,它们具备各种优异的物理化学特性。在样品前处理中,其可以与目标物通过多种作用力结合,拥有良好的萃取性能。但是碳基材料存在选择性差的缺点,研究人员通常利用对碳基材料的改性,引入-COOH、-OH、-NH_3_等官能团,或是与其他材料形成复合材料,以此来改善其萃取选择性^[6]^。

石墨烯是由*sp*^2^杂化连接的碳原子紧密堆积成蜂窝状的单层二维(2D)晶格结构材料,具有较高的比表面积和大*π*电子体系,是一种表面易修饰的高性能材料。Shen等^[7]^用石墨烯作为PT-SPE的萃取材料,并结合超高效液相色谱-串联质谱法(UPLC-MS/MS),建立了包括虾夷扇贝毒素(YTX)、冈田海绵酸(OA)、鳍藻毒素(DTX)等7种亲脂性藻毒素的分析方法,其检出限(LOD)和定量限(LOQ)均小于1.5 μg/kg和3.9 μg/kg。对来自中国6个沿海城市的67个贻贝样品进行了分析,结果表明OA为主要污染物,YTX含量较低。Liu等^[8]^采用共沉淀法合成了磁性石墨烯离子液体复合材料,将其用于MSPE,并结合UPLC-MS/MS,建立了微囊藻毒素(MCs)的分析方法。该方法具有良好的线性范围(0.005~10.0 μg/L)、低LOD(0.414~0.216 ng/L)、满意的回收率(83.6%~100.9%)和精密度(RSD < 7.59%),并在实际水样中检测到微量的MCs(0.003~0.021 μg/L)。GO表面和边缘含有大量的-OH、-COOH等极性官能团,可通过这些官能团对GO进行改性,或是借助其他纳米材料^[9]^、离子液体(ILs)^[10]^、聚合物^[11]^等对其进行功能化,继而改善其对目标物的萃取选择性。Tang等^[12]^利用聚乳酸-羟基乙酸共聚物修饰的GO纤维对活体动物进行快速采样(15 min),成功地将该纤维用于河豚外轴肌中河豚毒素(TTX)的萃取,LOD为0.52~2.30 ng/g,准确度良好。同时,CNT作为一种新型的萃取材料,由于其独特的结构和物理化学性质,如高孔隙率、中空结构、能够与分子形成*π-π*和范德华力等多重相互作用,并且易于功能化,在分析化学中得到了广泛应用^[13]^。Chen等^[14]^通过一步碳化法合成磁性金属/氮掺杂碳纳米管(M-NCNTs)用于萃取海水中的OA,随后使用高效液相色谱-串联质谱法(HPLC-MS/MS)进行分析。M-NCNTs拥有较大的比表面积、适当的孔结构和丰富的吡啶氮环,使其表现出萃取速率快(10 min)、吸附容量高(223.5 mg/g)等特点。在最佳条件下,该方法在1.0~800.0 pg/mL范围内呈现良好的线性关系(*r*=0.9992), LOD为0.4 pg/mL。最后,该方法成功用于海水样品的分析。Wei等^[15]^设计并制备了一种新型的CNT中空纤维膜,该纤维膜具有独特的非均相结构,不仅表现出较高的水通量和固有的筛分功能外,还具有将吸附和电化学氧化协同集成到简单膜过滤系统中的能力。因此,通过在纤维膜上的吸附和电化学氧化之间的方便切换,它们能够在高渗透通量下低成本地持续净化被MCs污染的水。

此外,其他碳基材料也具有优异的物理化学性质。Li等^[16]^研究表明生物炭与MCs的羧基、胍基之间具有氢键作用,这使其对MCs表现出良好的吸附性能,但由于材料与目标物间结合太紧密,导致MCs很难被洗脱下来。Teng等^[17]^基于双峰型介孔碳,通过化学修饰合成了具有高比表面积(1063 m^2^/g)和大孔容(0.7 cm^3^/g)的氨基功能化有序介孔碳,与未修饰的介孔碳材料相比,单位表面积的吸附容量增加了一倍。将其填装成SPE柱后,对MCs表现出优异的吸附性能(334 mg/L)、快速吸附行为和良好的再生性能。Huang等^[18]^使用浓盐酸处理介孔石墨氮化碳(mpg-C_3_N_4_),得到质子化介孔石墨氮化碳(mpg-C_3_N_4_-H^+^),该材料对MCs表现出高吸附容量(≥2000 μg/g),并且具有良好的稳定性及优异的重复使用性能。Park等^[19]^研究了几种介孔碳对于MCs的吸附,发现具有8.5~14 nm孔径的介孔碳对MCs的吸附容量最高,可应用于饮用水中低浓度MCs的高效萃取。

综上所述,碳基材料具有优异的稳定性及良好的吸附性能,通过改性后可以提高其萃取性能,能有效用于实际样品中藻毒素的高效萃取。

## 2 金属有机骨架材料

金属有机骨架材料(MOFs)是由金属离子与有机配体通过自组装形成的一类多孔材料。相对于传统无机材料,其具有更高的比表面积和孔隙率,还有更多样的结构和功能,并具有孔道规则和孔径可调的特点,使其被广泛应用于催化、吸附分离、气体存储、传感器等领域^[20]^。

近年来,MOFs在固相萃取领域的研究得到较大发展,特别是在环境监测方面。Wei等^[21]^合成了经吡啶接枝的锆基MOF(MIL-101(Cr)-Py)。由于在MIL-101(Cr)引入了吡啶,该MOF增强了与MCs之间的静电相互作用、*π*-*π*堆积相互作用和偶极-偶极相互作用,使其最大吸附容量(409.8 mg/g)远高于未修饰的MIL-101(Cr)。与HPLC结合后,建立了水体中MCs的高灵敏分析方法。Zhou等^[22]^合成了一种由MOFs和壳聚糖相结合的新型介孔材料(MOF-5@CS),并将其应用于水和鱼类样品中MCs和节球藻毒素(NOD)的高效萃取。该方法对MCs和NOD的LOD为0.0018~0.077 ng/mL,与一些针对复杂基质中MCs和NOD的检测方法相比,该方法对MCs和NOD的吸附性能更好,灵敏度也得到了极大提高。

只有少数的MOFs材料包含有磁性的金属中心,探究非金属中心MOFs的磁性功能化,将有助于扩大MOFs材料在MSPE领域的应用^[23]^。Zhang等^[24]^合成了磁性MOF核壳微球(Fe_3_O_4_@SiO_2_@UiO-66),首次作为MSPE萃取材料用于贝类样品中软骨藻酸(DA)的高效萃取,结合HPLC-MS/MS,成功建立了一种快速、简单而灵敏的DA测定方法。该方法分析时间短,线性良好(*r*=0.9995), LOD低(1.45 pg/mL),萃取重复性好(RSD≤5.0%, *n*=5),并成功在实际贝类样品中检测到了痕量的DA。Li等^[25]^合成了核壳结构的铜基磁性MOF复合材料(Fe_3_O_4_@PDA@Cu-MOFs)。Fe_3_O_4_@PDA@Cu-MOFs具有超高的比表面积、较强的磁响应和优异的亲水性。Li等将其与基质辅助激光解吸/电离-飞行时间质谱(MALDI-TOF-MS)相结合,用于实际水样中MCs的含量分析。在最佳的条件下,该方法在0.05~4.00 μg/L范围内具有良好的线性关系,在低水平(0.015 μg/L)下也具有良好的检测性能。该方法还成功地用于分析痕量MCs,对实际水样的定量回收率为98.67%~106.15%。Huang等^[26]^制备了一种磁性MOF材料(Fe_3_O_4_@ZIF-8/Zn^2+^),用于从复杂的生物基质中萃取DA。DA在Fe_3_O_4_@ZIF-8/Zn^2+^表面通过强静电作用和与不饱和锌离子的螯合作用快速吸附(约5 min)。利用Fe_3_O_4_@ZIF-8/Zn^2+^作为MSPE萃取材料,与HPLC-MS/MS相结合,得到了一种简便、快速、高效、灵敏的水产品中痕量DA的检测方法。该方法具有满意的精密度(RSD≤3.4%, *n*=5),线性范围为1.0~1000.0 pg/mL(*r*=0.9998),检出限为0.2 pg/mL,加标水产品的回收率为93.1%~102.3%。在实际贝类样品的分析中,该方法成功检测到微量DA(49.2 pg/mL),证明了Fe_3_O_4_@ZIF-8/Zn^2+^材料在水产品中检测DA的适用性。

MOFs具有比表面积高、活性位点丰富等优点,但大多数MOFs缺乏选择性,特别是对极性或亲水性的藻毒素,疏水MOFs材料的界面相互作用和吸附效率会受到抑制。因此,Xu等^[27]^利用静电纺丝技术,以及MOFs种子生长和金纳米颗粒(AuNPs)连接适配体策略,制备了一种新型静电纺丝纳米纤维涂层(Apt@AuNPs@UiO-66-NH_2_),用于特异性萃取MCs。将负载有MOFs和适配体的纳米纤维应用于SPME,并与液相色谱-质谱(LC-MS)相结合,实现了实际水体样品中MCs的选择性萃取,且几乎没有受到类似物干扰。次年,Xu等^[28]^还提出了一种拥有更高适配体覆盖密度的新型适配体功能化金属有机骨架纳米纤维复合材料(PAN/UiO@UiO-N_3_-适配体),并将其用于MCs的选择性萃取。该方法具有良好的选择性、稳定性和重现性,检出限为0.003 ng/mL,优于大多数非特异性的SPME纤维或SPE柱。

近年来,由MOFs衍生化制备的多孔材料也受到了广泛关注。它们在拥有高比表面积的同时,还可以保留其前驱体MOFs的原始形貌、孔隙率和化学成分^[23]^。Chen等^[29]^通过直接碳化法合成了基于钴(Ⅱ)-MOF的磁性氮掺杂碳纳米管笼(N-CNTCs)。在碳化过程中,MOF内的钴离子转化为N-CNTCs的磁性功能纳米颗粒。同时,来自有机配体的大量氮被掺杂到碳框架中。这种独特的结构使N-CNTCs具有优异的化学稳定性、高亲和力和良好的分散性。合成的磁性N-CNTCs作为MSPE萃取材料,与HPLC-MS/MS相结合,建立了一种简便、高效、灵敏的OA检测方法。该方法的线性范围为3.0~1000.0 pg/mL,线性良好(*r*≥0.9997), LOD和LOQ分别为1.3 pg/mL和3.0 pg/mL。采用该方法对几种海鲜样品进行了分析,表现出良好的回收率(82.0%~107.0%)和重现性(RSD≤4.5%)。

MOFs不但具有极高的孔隙率、可调节的孔径大小,还具有丰富的结构和功能,可以根据目标物的理化性质来进行合理的设计。此外,与其他材料结合形成新型复合材料及其自身的衍生化材料,其在保留自身优点的同时,还能获得更优异的性能。虽然MOFs在样品前处理领域备受关注,但仍有许多的难点需要解决,例如功能单体合成工艺复杂、生产成本高、量产困难等。

## 3 共价有机骨架材料

共价有机骨架材料(COFs)是一类由有机结构单元以共价键自组装形成的多孔结晶材料^[30⇓-32]^。由Yaghi等^[33]^在2005年基于网状拓扑学原理率先制备。COFs通常是由C、H、O、N、B等轻质元素组成,可以根据形成的共价键类型,分为硼酸酐类、硼酸酯类、席夫碱类、三嗪类、亚胺类、腙类等,具有良好的发展前景。COFs具有结晶度及结构稳定性好、密度低、比表面积高、结构可设计等特点,被广泛应用于吸附与分离、能源、催化等诸多领域^[34]^。

COFs萃取能力主要取决于材料的拓扑结构以及构成骨架的有机单体,可以提供*π*-*π*堆积、静电、亲/疏水、氢键等相互作用^[35]^。Salonen等^[36]^制备了一种在水中具有良好稳定性的COF材料(TpBD-Me_2_),并评估了其对OA的吸附性能。由甲基化有机单体构成的TpBD-Me_2_具有疏水性的空腔,更有利于吸附疏水性的OA,因此TpBD-Me_2_COF对OA的吸附量是常规树脂的38倍^[37]^,并可以更快达到吸附平衡。Fernandes等^[38]^研究了3种COFs材料(TpBD-(CF_3_)_2_、TpBD-(NO_2_)_2_和TpBD-(NH_2_)_2_)对MCs的吸附性能。研究结果表明,TpBD-(NO_2_)_2_对含有精氨酸的MCs吸附效果最佳,而TpBD-(NH_2_)_2_能够高效萃取含有丙氨酸的MCs。Qi等^[39]^开发了一种新颖的三维COFs(3D-COF),其孔壁上具有可调控的活性羟基,使其同时具有亲水和疏水相互作用,可用于OA的高效萃取。与之前报道的无亲水相互作用的萃取材料相比,该材料在萃取微量OA方面得到了显著提高。Wang等^[40]^通过2,4,6-三甲酰基苯醇(Tp)与2,5-二氨基苯甲酸(Pa-COOH)自组装制备了一种*β*-酮亚胺基COFs材料(TpPa-COOH)。该羧酸功能化的TpPa-COOH在萃取性能上优于含甲基的COFs材料(TpPa-CH_3_)。TpPa-COOH对石房蛤毒素(STX)的吸附速度快,在1 h内能达到平衡状态,且最大吸附容量为5.69 mg/g。更重要的是,该TpPa-COOH材料还显示出良好的可重复使用性和在天然水体中对STX的高回收率。

此外,COFs材料的形貌特征也对藻毒素的高效萃取具有一定影响。Yu等^[41]^在室温下合成了一种具有花瓣状结构的空心COFs(HFH-COF)。所合成的HFH-COF不仅具有介孔结构、非常大的比表面积、优异的化学稳定性和良好的晶体度,并且特殊的空心形态赋予其高表面积利用率和快速传质速率。与球形COF(S-COF)比较,HFH-COF可以更快达到萃取平衡,具有更好的萃取性能。随后,与HPLC-MS/MS相结合,建立了一种MCs的高灵敏检测方法。该方法具有LOD低(0.6~0.8 pg/mL)、线性范围宽(1.5~1000.0 pg/mL)、重复性良好(RSD≤7.6%, *n*=6)等特点。

由于COFs自身没有磁性,为将COFs作为MSPE萃取材料,需要开发具有磁性的COFs复合材料。Romero等^[42]^报道了一种制备磁性COFs的新方法。该方法利用多巴胺可将Fe_3_O_4_颗粒迅速氨基功能化,随后在氨基化的Fe_3_O_4_颗粒表面外延生长COFs材料(mTpBD-Me_2_)。合成的复合材料首次用于藻毒素的高效萃取,对OA和DTX的最大吸附量分别为812和830 mg/g。与常用的无磁性大孔树脂相比,对OA的吸附量增加了500倍,对DTX的吸附量增加了300倍。Cao等^[43]^采用一步法在室温下合成了一种核壳结构的磁性COFs材料(Fe_3_O_4_@TaTp),将其用于海水和贝类中OA的萃取,并与HPLC-MS/MS相结合,建立的分析方法具有良好的线性(*r*≥0.9994)、满意的准确度和精密度(RSD ≤15%)、低LOD(海水0.5 pg/mL,贝类0.04 μg/kg),并成功用于实际样品的分析。Liu等^[44]^通过机械研磨法快速制备了一种磁性CNT与COFs的复合材料(MCNTs@TpPa-1),并将其作为MSPE萃取材料用于MCs的高效萃取。结合HPLC-MS/MS,建立了一种水样中MCs的高灵敏分析方法。该方法具有良好的重复性(RSD≤6.8%)、可靠的线性(*r*≥0.9991)和宽线性范围(2.0~1000 pg/mL)。此外,还利用所开发的方法成功地在湖泊水样中检测出痕量MCs(9.6~24.6 pg/mL)。Wang等^[45]^通过一锅法合成了一种含有芘结构的磁性COFs材料(Fe_3_O_4_@Py-COF)。Fe_3_O_4_@Py-COF具有高比表面积、丰富的纳米孔隙和良好的稳定性,同时表现出对MCs良好的吸附能力和萃取效率。与高效液相色谱-二极管阵列检测法(HPLC-DAD)相结合,在10~1000 μg/kg范围内获得了良好的线性关系(*r*≥0.9994), LOD为3.0~4.5 μg/kg,且RSD低于4.9%(*n*=5)。

相对于MOFs, COFs拥有更加稳定的结构,并且也可以根据目标物进行适当的功能化。这些优点为其在样品前处理领域的应用奠定了良好的基础。

## 4 分子印迹聚合物

分子印迹聚合物(MIPs)是拥有选择性萃取能力的最具吸引力的材料之一^[46,47]^,在该技术中,交联剂、聚合引发剂、模板分子和功能单体(与模板分子相互作用)同时聚合,然后,从所制备的聚合物中去除模板分子,继而实现基于印迹效果的选择性萃取。MIPs由于具有成本低、选择性高、通用性强等优点在食品和生物分析领域得到了广泛应用^[48⇓-50]^。

在该技术中,模板分子是赋予MIPs选择性的关键,而藻毒素标准品的价格高昂,直接利用标准品作为模板分子是不切实际的,因此筛选适当的伪模板分子是首要任务。Zhou等^[51]^以1,3,5-戊三羧酸为伪模板分子,所制备的MIP对DA具有较高的亲和力,能选择性萃取水产品中的DA,并建立了相应的HPLC分析方法,线性范围为0.5~25 mg/L(*r*≥0.9950),贻贝提取液中DA的加标回收率为93.4%~96.7%。Lin等^[52]^同样以1,3,5-戊三羧酸为伪模板分子,通过乳液聚合法制备了选择性萃取DA的MIP。该聚合物对DA具有较高的亲和力,解离常数为13.5 μg/mL,最大吸附量为1249 μg/g。与HPLC结合所建立的分析方法的LOD为0.17 μg/g,加标回收率为93.0%~98.7%。结果表明,利用该伪模板分子制备的MIPs可用于复杂样品中DA的选择性萃取。同年12月,Lin等^[53]^继续将Fe_3_O_4_纳米粒子引入到聚合物的制备中,得到一种用于选择性萃取DA的磁性MIP。该聚合物颗粒不仅具有高磁化强度,可用于快速磁性分离,而且拥有良好的表观吸收率(1600 μg/g)和解离常数(20.6 μg/mL)。同时,该MIP重复使用5次后,还能保持90%的吸附量。将其作为MSPE萃取材料用于贝类中DA的HPLC检测,LOD为0.050 μg/g。Elbert等^[54]^以L-精氨酸为伪模板分子,以甲基丙烯酸(MAA)为功能单体,二甲基丙烯酸乙酯(EDMA)为交联剂,酸化乙腈为前原体,2,2-偶氮(2,4-二乙基戊腈)为引发剂,制备了一种羧基功能化MIPs,能选择性识别含精氨酸的MCs,回收率高达70.8%~91.4%。Mei等^[55]^以鸟苷为模板分子,*α*-甲基丙烯酸为功能单体,乙二醇二甲基丙烯酸酯为交联剂,通过本体聚合合成了一种新型MIP。该MIP对膝沟藻毒素(GTX)具有较高的选择性。所建立的高效液相色谱-荧光检测(HPLC-FLD)对加标海水样品的回收率可达88%,方法的线性范围为0~200 μg/L(*r*≥0.9950), LOD为0.5 μg/L,并成功地从微小亚历山大藻和塔马亚历山大藻中分别检测到GTX(0.99~1.10 μg/L)。Lian等^[56]^以咖啡因和己酮可可碱为模板,采用双模板印迹技术制备了新型MIP微球。合成的聚合物对GTX具有良好的亲和性,并被用作SPE萃取材料,对GTX的萃取效率可达73.2%~81.5%。通过与HPLC-FLD结合,建立了一种小麦草样品中GTX的高灵敏分析方法,能有效去除干扰基质。Zhang等^[57]^以咖啡因为伪模板分子,制备了一种新型的MIP材料,作为SPE柱填料,加标回收率为62.8%~105%,并与HPLC-MS相结合,建立了海水中GTX的检测方法,其检出限为47.4 ng/L。

此外,也有少量研究直接选用目标藻毒素作为模板分子。Tian等^[58]^在磁性GO表面成功合成了一种新型磁性分子印迹聚合物(GO@Fe_3_O_4_-MIP),用于高选择性萃取水样中含精氨酸的MCs。GO@Fe_3_O_4_-MIP不仅具有较高的吸附能力,最大吸附量可达1.24 mg/g,且吸附速率快(25 min)。与HPLC结合建立的分析方法在2~10000 μg/L范围内线性良好(*r*=0.9985), LOD为0.08 μg/L,加标回收率为86%~113%。Pan等^[59]^同样以GO为基底,制备了双面磁性分子印迹聚合物(DS-MMIP@GO),双面分子印迹聚合物修饰结构为DS-MMIP@GO提供了丰富的吸附位点,使其对痕量级MCs表现出优异的萃取效率和选择性。在最佳萃取条件下,对MCs的富集因子能达到2000。该方法对MCs的LOQ为0.1~2.0 ng/L,线性范围为0.1~500 ng/L,回收率为84.1%~98.2%, RSD小于10%。

MIPs是一类优异的高选择性萃取材料,它的活性位点可以特异性地吸附目标分子,在样品前处理方面已得到广泛应用。新型的MIPs及其应用的不断改进将会推动样品前处理技术在藻毒素分析检测领域的不断发展。

## 5 其他萃取材料

除了以上萃取材料之外,近年来也有一些其他新型材料开始应用到藻毒素的萃取中。其中,多数萃取材料是以SiO_2_为基底进行制备的。Sun等^[60]^制备了一种铜离子修饰的磁性介孔硅纳米颗粒(Fe_3_O_4_@mSiO_2_@Cu^2+^),其具有均匀的粒径大小、良好的磁性能和特异的选择性。将所合成的Fe_3_O_4_@mSiO_2_@Cu^2+^作为MSPE萃取材料,与HPLC相结合,发展了一种测定水样品中痕量MCs的高灵敏方法。该方法在0.1~15 μg/L范围内呈现良好的线性(*r*=0.9994),检出限为0.025 μg/L。Li等^[61]^通过水热合成法制备了一种由溴化十六烷基吡啶修饰的磁性硅球(Fe_3_O_4_@SiO_2_@CPC)。通过优化萃取材料的用量、萃取时间、洗脱溶剂比例和洗脱体积等条件,实现了对水样中MCs的高效萃取。该研究所使用的磁性萃取材料具有制备简单、价格低廉、易于固液分离等优点,并可用于对环境水样中MCs的高灵敏检测。Xu等^[62]^报道了一种新的反相/弱阴离子交换混合模式的磁性介孔硅球(Fe_3_O_4_@nSiO_2_@mSiO_2_@C_18_@NH_2_),用于分析贝类中6种亲脂性藻毒素(AZAs和OA及其衍生物)。该萃取材料对分析物具有优异的吸附能力和选择性,能显著减少复杂基质所带来的干扰。所提出的方法在工作范围内对AZAs(2.0~200.0 ng/mL)和OA及其衍生物(4.0~200.0 ng/mL)表现出良好的线性相关度(*r*为0.9980~0.9991)、令人满意的回收率(82.8%~118.6%)、较低的LOD(0.4~1.0 μg/kg)。Devasurendra等^[63]^报道了一种金-聚吡咯纳米复合物包覆的二氧化硅材料(SiO_2_@Au-PPy),用于高效萃取6种常见MCs。该材料对所有MCs的平均回收率可达103.2%, RSD为1.6%~5.4%,表明其具有良好的萃取效率和重现性,是一种有望用于水样中MCs定量分析的新萃取材料。

同时,也有利用硼酸与邻二醇相互作用的萃取材料。Mudge等^[64]^开发了一种硼酸亲和聚合物用于选择性萃取含有邻二醇的西加鱼毒素(CTXs)。该硼酸亲和聚合物在1 h内可实现对鱼提取物中CTXs的高效萃取,回收率可达95%。这种选择性萃取材料在CTXs的制备和分离方面有很大的应用潜力。Mile等^[65]^报道了使用硼酸凝胶从贻贝提取物中选择性地萃取原多甲藻酸毒素(AZAs)的方法。该硼酸凝胶对大多数AZAs类似物都具有良好的回收率,并且还能够有效消除后续HPLC-MS/MS分析过程中可能出现的基质效应。

此外,还有利用金属亲和作用、主客体相互作用等的萃取材料。Zhang等^[66]^制备了一种铁氧体磁性纳米球(CuFe_2_O_4_),并将其作为MSPE萃取材料,用于DA的高效富集和去除基质中盐的干扰。与HPLC-MS/MS结合所开发的分析方法在5~1000 pg/mL范围内显示出良好的线性关系(*r*=0.9995),低LOD(2.5 pg/mL),良好的回收率(86.0%~98.1%)和可接受的重复性(RSD≤6.5%, *n*=3)。Huang等^[67]^制备了一种磁性*γ*-环糊精聚合物(Fe_3_O_4_@PDA@*γ*-CDP),并将其作为MSPE萃取材料,用于MCs的高效富集。所制备的Fe_3_O_4_@PDA@*γ*-CDP在水中具有良好的分散性和对MCs高度的亲和力。结合HPLC-MS/MS,开发了一种方便、灵敏且高效的方法用于检测水样中的MCs,该方法在1.0~1000 pg/mL范围内呈良好线性关系(*r* ≥0.9992)和低LOD(0.8~2.0 pg/mL)。最后,该方法成功应用于实际样品中MCs的检测。Li等^[68]^通过一种简便的方法合成了L-半胱氨酸修饰的磁性金属-有机配合物(Fe_3_O_4_@L-Cys),并应用于大鼠血浆样品中STX的磁性固相萃取,随后进行HPLC-MS/MS检测分析。所开发的方法在5~300 ng/mL范围内表现出良好的线性关系(*r*=0.9992), LOD为0.5 ng/mL。

## 6 结论与展望

新型萃取材料的研究是藻毒素分析检测的一项重要内容。近年来,备受关注的萃取材料包括碳基材料、COFs、MOFs、MIPs等。这些新型萃取材料因其优异的性能和独特的微观结构,被广泛应用于复杂环境样品、生物样品,以及食品样品中目标物的分离分析,是藻毒素萃取的主要材料。目前,虽然这些新型萃取材料在藻毒素前处理方面都发挥着不可替代的作用,但是它们都存在一定的不足:(1)碳基材料、MOFs和COFs具有表面积高、吸附位点丰富等优点,但它们对目标物的选择性吸附能力相对较差;(2)大多数MOFs在水相中不稳定,难以用于含水溶液样品中的萃取;(3)COFs主要由轻质元素组成,质量很轻,较难通过离心力与样品溶液完全分离,限制了其应用范围;(2)MIPs虽具有较好的选择性,但模板分子流失、传质速度较慢、吸附容量偏低等问题亟待解决。因此,针对藻毒素的特性来合理设计、制备新型功能化萃取材料,以及研究新型复合萃取材料是极具应用潜力的方向。

此外,每个样品前处理技术都有各自的优缺点,针对不同种类的藻毒素、不同基质的样品特性,选择合适的前处理技术进行联用,是富有挑战性和前景的发展方向。不同技术的联用不仅有助于提高复杂样品中藻毒素的提取效率,而且能有效除去基质中的各类干扰物,如脂类物质、色素等。综上所述,新型萃取材料在藻毒素分离分析领域的发展和应用还有很多可探索的空间。
